# Validation of a Pediatric Primary Care Network in a US Metropolitan Region as a Community-Based Infectious Disease Surveillance System

**DOI:** 10.1155/2011/219859

**Published:** 2011-12-07

**Authors:** Kristen A. Feemster, Yimei Li, Robert Grundmeier, A. Russell Localio, Joshua P. Metlay

**Affiliations:** ^1^Division of Infectious Diseases, Department of Pediatrics, The Children's Hospital of Philadelphia, Perelman School of Medicine, University of Pennsylvania, Philadelphia, PA 19104, USA; ^2^Robert Wood Johnson Foundation Clinical Scholars Program, Perelman School of Medicine, University of Pennsylvania, Philadelphia, PA 19104, USA; ^3^Leonard Davis Institute of Health Economics, University of Pennsylvania, Philadelphia, PA 19104, USA; ^4^Division of Oncology, The Children's Hospital of Philadelphia, Philadelphia, PA 19104, USA; ^5^Center for Clinical Epidemiology and Biostatistics, Perelman School of Medicine, University of Pennsylvania, Philadelphia, PA 19104, USA; ^6^Center for Biomedical Informatics, The Children's Hospital of Philadelphia, Philadelphia, PA 19104, USA

## Abstract

This cross-sectional study used Geographic Information System methods to compare sociodemographic and clinical characteristics of children enrolled and not enrolled in a primary care network to determine the suitability of the network to estimate population-based disease rates. We validated the network surveillance system by comparing invasive pneumococcal disease rates between network and nonnetwork children using population-based surveillance data. Among the study population of 130300 children, network children were more likely to be female, Black, non-Hispanic, younger, and receive Medicaid. These differences varied across neighborhoods, however, adjusting for neighborhood characteristics did not significantly change observed differences. Rates of invasive pneumococcal disease were not significantly different between network and non-network children. Significant demographic and clinical differences existed between network and non-network children and varied over small areas. Observed population rates of an infectious disease did not significantly differ suggesting that the network can potentially provide valid disease estimates for the community population.

## 1. Introduction

Population-based infectious diseases surveillance is essential for epidemiological investigation, disease tracking, and public health planning. Current sources of data include voluntary physician reporting through syndromic surveillance programs, mandatory reporting through local and state health departments, and sentinel provider networks [1–6]. However, accurate population-based sampling is challenging, particularly for common infectious diseases that, in some countries, do not typically present to acute care facilities. The emergence of primary care networks, linked to primary care practice sites within defined regions, has the potential to play an important role in infectious disease surveillance [7–10]. An outpatient primary care network may provide more complete capture of disease entities that are often mild and do not require emergency room or inpatient care and the increased use of electronic medical records provides more readily available data. Despite this potential, there are few studies evaluating the performance of community-based surveillance within primary care networks, especially pediatric primary care networks.

The use of a primary care network to sample community members for disease prevalence estimates assumes that the patients seeking care in the network are an unbiased sample of the source community. Representativeness may be assumed or based upon comparisons to national population samples [7, 10–16]. Examples of community-level comparisons include an evaluation of a sentinel primary care network used for influenza surveillance by comparing demographic characteristics of their practice population with the general population in their practice areas [11]. These results showed that the network underestimated patients from areas of deprivation and recommended the recruitment of additional sites. Other researchers have attempted to determine methods to “harmonize” potential group level confounding factors such as insurance status or care-seeking behavior so that conclusions can be drawn about patterns of disease from practices [17].

The primary objective of this study was to use geographic information system- (GIS-) based methods to compare the characteristics of children seeking care within a pediatric primary care network with those of children in the source neighborhoods as a first step in determining the suitability for such a network to conduct infectious diseases surveillance [18, 19]. Specifically, we measured whether children who seek care within the network reflect the sociodemographic and clinical characteristics of the pediatric population of the metropolitan region served by these practices. Secondarily, we determined the neighborhood characteristics that were associated with the level of clinical and demographic variation between the network patient population and the pediatric population of the neighborhoods within the metropolitan region. We hypothesized that sociodemographic and clinical characteristics of the patients receiving care from the pediatric primary care network would approximate the characteristics of the service region pediatric population, but that there would be significant variation in the degree of similarity at the neighborhood level.

## 2. Methods

### 2.1. Study Design

This cross-sectional study compared sociodemographic and clinical characteristics of children enrolled in the Children's Hospital of Philadelphia (CHOP) primary care network within the metropolitan Philadelphia (network children) versus the remaining children not enrolled in the network residing in the same region (nonnetwork children). The CHOP primary care network is comprised of 29 practices in metropolitan Philadelphia and New Jersey representing approximately 600000 yearly visits for 125000 children. A cohort of 128985 children who resided within the metropolitan Philadelphia region and received healthcare at least once from a network practice between 1 November 2004 and 31 October 2006 were included in this study. We defined the metropolitan Philadelphia region as the five Pennsylvania counties surrounding the city: Bucks, Chester, Delaware, Montgomery, and Philadelphia. All patients from the CHOP practices in New Jersey or Delaware or who resided outside of the five-county region were excluded from the study as population estimates for nonnetwork children were not available for these regions.

### 2.2. Data Sources

Data on characteristics of nonnetwork children (≤18 years) were obtained from the Philadelphia Health Management Corporation's (PHMC) 2006 Community Health Southeastern Pennsylvania Household Health Survey, a major telephone survey of more than 10000 households (13,000 adults and children) that examines the health and social well-being of residents in Bucks, Chester, Delaware, Montgomery, and Philadelphia counties. The survey is conducted as part of PHMC's Community Health Data Base, which contains information about health status, use of health services, and access to care. PHMC is a nonprofit, public health organization committed to improving the health of the community through outreach, education, research, planning, technical assistance, and direct services [20].

This database contains individual level data collected as part of a probability sample of families from the five-county metropolitan Philadelphia region. Data regarding children is collected through interviews of a child proxy or an adult in the household with knowledge regarding the child's health. The survey is performed every two years and also oversamples specific demographic groups. To generate population level estimates, the survey results were frequency weighted based upon the inverse of the demographic probability sampling distributions and adjusted for survey nonresponse rates. Specific data abstracted from the PHMC database included age, gender, race/ethnicity, diagnosis of asthma and diabetes, insurance type, residential census tract, and residential neighborhood [20].

Data of network children (≤18 years) were extracted from the CHOP electronic health record (EpicCare, Epic Systems Corporation, Verona, WI). The electronic health record includes clinical and sociodemographic information, family history, and residential address. Sociodemographic characteristics included gender, age, race/ethnicity, insurance type, number of clinic visits in the past year, and residential block address. Clinical characteristics included diagnosis of asthma and diagnosis of diabetes. Diagnosis of asthma was determined from problem list ICD9 codes and subcodes for asthma (493.x) and diabetes (250.x) [21].

### 2.3. Neighborhood Designation

We used minor civil divisions (MCD) to define neighborhood. An MCD is defined as “the primary governmental or administrative division of a county” and can be comprised of one to several census tracts [22]. This unit was utilized across all counties and corresponds to a town or township that approximates a historically defined community sharing similar characteristics and resources. The PHMC database included geocoded data of all households to a census tract and MCD. Utilizing the residential address provided from the EPIC database, all CHOP patients were also geocoded to census tract and MCD. Geocoding was performed through the Cartographic Modeling Laboratory at the University of Pennsylvania. Census tracts were aggregated to their corresponding minor civil divisions. We also calculated the linear distance between patient's residence and clinic utilizing ArcView 9.0 (ESRI, Inc., Redlands, CA). 

### 2.4. Defining the In-Network and Out-of-Network Populations

**Figure 1:**
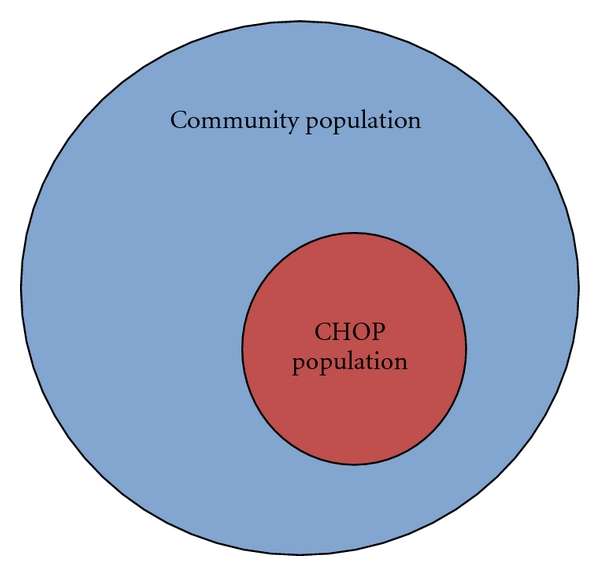
Conceptual framework for the study population.

Our conceptual framework was based upon the underlying assumption that the population of children seen within the primary care network and geocoded to the 5-county region was a representative subset of the larger population of children residing within the 5-counties (Figure 1). We used the PHMC results with their associated weights to calculate weighted totals for all subjects per neighborhood. We poststratified the survey weight based upon US census 2000 results so that the survey total sample weight by neighborhood equaled the census total. The primary care network population for each MCD calculated above was then subtracted from the reweighted survey totals for each MCD to obtain the number of children within each neighborhood who were not enrolled in the primary care network (nonnetwork population). The fraction of children in each MCD that were not enrolled in the primary care network was used to reduce the sampling weights for each subject in the community survey proportionally such that

(1) $$\begin{array}{cc} & \sum \text{new}\;\text{survey}\;\text{weights}\\ & \;\;*\text{PHMC}\;\text{data}\;\left(=\text{nonnetwork}\;\text{children}\right)\\ & \;\;+\sum \text{children}\;\text{in}\;\text{primary}\;\text{care}\;\text{network}\\ & \;=\text{total}\;\text{children}\;\text{in}\;\text{the}\;\text{region}\;\left(\text{based}\;\text{upon}\;\text{census}\;\text{data}\right). \end{array}$$

This provided two independent patient populations for comparison.

## 3. Data Analysis

### 3.1. Descriptive Statistics

For the primary care network population, we aggregated all patient level data from the eligible network practices and calculated means with standard errors for the proportion of each sociodemographic and clinical characteristic. To obtain population measures for metropolitan Philadelphia, we utilized PHMC data, incorporating the adjusted sampling weights described previously. We also aggregated both network and PHMC patient data by county and neighborhood.

### 3.2. Univariable and Multivariable Analysis

To determine the statistical significance of differences between network and nonnetwork children, we compared population prevalence of each characteristic and calculated unadjusted odds ratios. These analyses were performed across the total population then stratified by county and by neighborhood to determine whether or not there was regional and small-area variation in the degree of similarity between the network and nonnetwork patient populations. To determine the neighborhood characteristics that confounded the measured clinical and demographic differences between network and nonnetwork children, we performed multivariate analysis with logistic regression that incorporated the survey weights using PROC SURVEYLOGISTIC in SAS.

The patient-level-dependent characteristics evaluated were age group, gender, race/ethnicity, presence of asthma, presence of diabetes, and participation in Medicaid. The main independent variable of interest in each model was the child's enrollment status in the primary care network. The neighborhood-level covariates included in the multivariable models as potential confounders were average distance traveled by patients within a neighborhood to their practice, neighborhood average household size, average income of neighborhood, and, for Philadelphia County, density of pediatric providers within residential neighborhood. These analyses were performed across the total population and stratified by county.

### 3.3. Neighborhood Analyses

In addition to an overall five-county analysis, we also conducted separate analyses stratified by neighborhoods within Philadelphia. Philadelphia county is comprised of 45 neighborhoods (MCDs) which were combined into 13 larger neighborhoods that correspond to groups of neighborhoods recognized by civic organizations as areas sharing common characteristics and resources. We focused upon the patient characteristics that may affect risk for presenting with an infectious disease including age group, race/ethnicity, and Medicaid receipt. We performed unadjusted comparisons of each of these characteristics between network and nonnetwork children in each of the 13 Philadelphia neighborhoods. For each characteristic, we constructed forest plots to illustrate the unadjusted odds ratio and associated confidence intervals for each neighborhood.

### 3.4. Comparison of Infectious Disease Rates

As a specific test of the accuracy of the network to conduct infectious diseases surveillance, we compared rates of bacteremic pneumococcal disease among patients <18 years of age in the Philadelphia metropolitan region. We selected this indicator condition due to the availability of population-based surveillance data that was collected as part of a prospective surveillance program for bacteremic pneumococcal disease among adults and children in the Philadelphia metropolitan region from 2006 to 2009. Forty-eight of 49 of acute-care adult and pediatric hospitals in the Philadelphia metropolitan region participate in the surveillance network, and the nonparticipating hospital represents <5% of all cases in the region [23, 24]. As part of the surveillance network, parents of children with bacteremic pneumococcal disease were interviewed and reported the primary care site for the child, allowing us to assign the child as network versus nonnetwork. We compared the number of cases among network children to the number of cases among nonnetwork children. The number of network and nonnetwork cases was divided by the total number of children in the network and nonnetwork populations to obtain annual incidence rates which were compared using Poisson regression. We calculated unadjusted rate ratios and rate ratios adjusted for the following neighborhood characteristics: proportion of the population who is Black, proportion of the population receiving Medicaid, and proportion of the population <6 years of age.

Twenty-eight cases (of 108 cases) did not provide primary care network information. We conducted multiple imputations on these cases based upon their residence information. Specifically, we imputed a case's network status according to the probability of being in the CHOP network given residence in a particular neighborhood. Imputations were done in STATA using the MI procedure. We then repeated the above analysis on each of the 10 imputed data sets and combined the results by using PROC MIANALYSE in SAS to correctly account for the added variance from the imputed data.

All analyses were performed using STATA 11.0 (StataCorp LP, College Station, TX or SAS 9.2 (SAS Institute, Inc., Cary, NC). A *P* value of <0.05 was used to determine significance. The study was approved by the Institutional Review Boards at the Children's Hospital of Philadelphia and Perelman School of Medicine at the University of Pennsylvania.

## 4. Results

**Figure 2:**
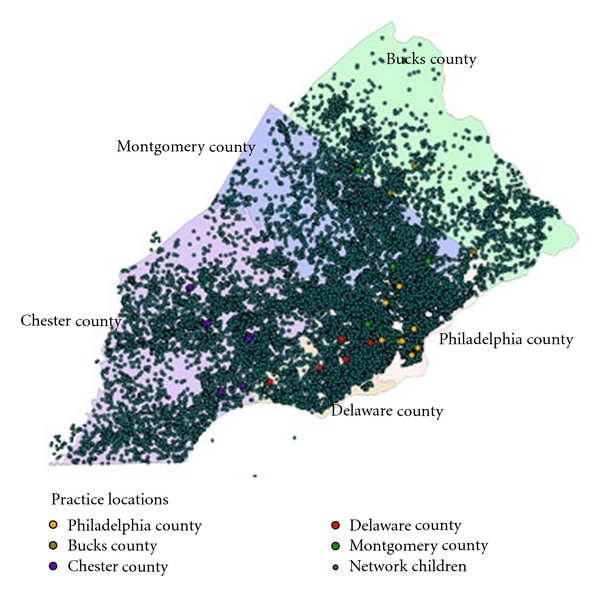
Distribution of patient population from the Children's Hospital of Philadelphia Primary Care Network.

A total of 132383 children were enrolled in the primary care network of which 128985 resided within the 5-county Philadelphia metropolitan region and were geocodable. There were 1768 duplicate records that were removed from the dataset for a total of 127217 network-enrolled children.  Figure 2 displays the home location of all children within the network in relation to all of the primary care sites within the network. Network children were widely distributed throughout the five-county metropolitan region. However, the majority of the sample resided within Philadelphia and Chester counties (42680 (32.8%) and 33617 (25.8%), resp.), and a higher proportion of network children resided in neighborhoods close to practice locations. There were 8550 (6.6%) network children who resided in census tracts that could not be matched to a defined neighborhood and were therefore not included in the neighborhood analyses. There were a total of 3083 children in the PHMC database, which, after adjustment of weights, resulted in a weighted total of 901655 nonnetwork children and a combined total of 1028872 network and nonnetwork enrolled children.

Comparing network and nonnetwork children, there were significant differences for all of the sociodemographic and clinical variables except for the prevalence of asthma (Table 1). A higher proportion of network children were female, Black, younger than 12 years of age, and receiving Medicaid. A lower proportion of network children was Hispanic and diagnosed with diabetes. When stratified by county, significant differences remained for age and race/ethnicity across all counties where children outside of the network were more likely to be Hispanic and older.

Results from the unadjusted logistic regression were similar: among the total population, the odds of being female, Black, and receiving Medicaid were higher for network children compared to children outside of the network, while nonnetwork children had a higher odds of being Hispanic and older (Table 2). Adjusting for neighborhood average household size, average income, average distance traveled by patients to their primary care practice, and density of pediatric providers within the neighborhood did not significantly change the observed differences between network and nonnetwork children.

### 4.1. Neighborhood Analyses

**Figure 3:**
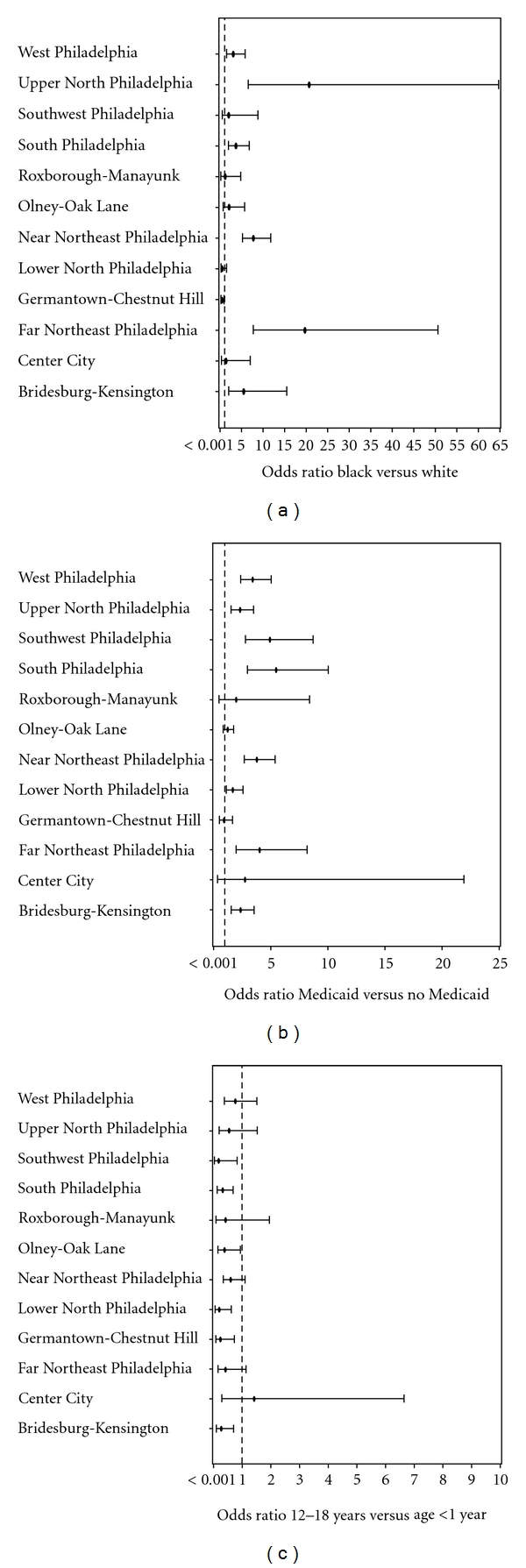
(a) Odds ratios for race (Black versus White) comparing the CHOP versus community populations by neighborhood, Philadelphia County. (b) Odds ratios for Medicaid (Medicaid versus no Medicaid) comparing the CHOP versus community populations by neighborhood, Philadelphia County. (c) Odds ratios for age (12–18 years versus <1 year old) comparing the CHOP versus community populations by neighborhood, Philadelphia County.

We next examined the differences between network and nonnetwork children for race/ethnicity, Medicaid receipt, and age stratified by neighborhood within Philadelphia. Overall, the point estimates for differences between network and nonnetwork children were smaller in each Philadelphia neighborhood, but there was variation across neighborhoods. Selected comparisons to illustrate regional differences are presented in Figure 3. Network children were more likely to be Black (versus White) in South and West Philadelphia as well as North and Northeast Philadelphia and more likely to be covered by Medicaid in all neighborhoods except for Roxborough and Germantown. Network children were less likely to be older (12–18 years old) in all neighborhoods except for the North, Northeast, Center City, and West Philadelphia neighborhoods.

### 4.2. Comparison of Infectious Disease Rates

There were a total of 108 cases of invasive pneumococcal disease among the pediatric population in the Philadelphia metropolitan region identified from a population-based surveillance hospital network from October 2006 to September 2009. Of these, 18 cases were identified within network children and 62 cases within nonnetwork children. The remaining 28 cases did not provide primary care network information and required imputation based upon residential neighborhood to assign network enrollment status. The estimated annual incidence rates were 18 cases per 100000 children among the network population and ten cases per 100000 children among the nonnetwork population, a difference that was not statistically significant (IRR 1.69, 95% C.I. 0.72, 3.96).

## 5. Discussion

In this paper, we investigated the potential for a pediatric primary care network to perform disease surveillance among the community population served by the network by measuring whether the network patient population was representative of the overall community pediatric population. While other studies describe sentinel surveillance networks and their representativeness, to our knowledge this is the only study to systematically evaluate a pediatric primary care network in this manner [7, 10–15]. Our results demonstrate that the children within the primary care network were widely distributed across the five-county Philadelphia metropolitan region, but, as expected, network children were more likely to reside closer to practice locations. We observed significant demographic and clinical differences between network and nonnetwork children, indicating that population-based surveillance estimates derived from this network might be biased. However, the observed differences were not consistent across counties and neighborhoods, suggesting that the representativeness of the primary care network varied over smaller areas. Such information could inform the use of the primary care network for infectious diseases surveillance in the future both by highlighting areas with substantial biased sampling and, possibly, identifying methods for adjusting estimates to more closely approximate the source population. Interestingly, despite these measured differences, our analysis of a specific population-based infectious disease rate (pneumococcal bacteremia) suggests that the estimate provided by the primary care network was not significantly different from the true underlying population rate.

 The most persistent difference between the network and nonnetwork children, even in the stratified analyses, was the probability of being Hispanic and the probability of being older. The proportion of Hispanic children and children aged 12–18 was significantly higher in the nonnetwork enrolled population. This is different from findings from one study comparing patients from a practice-based research network and national comparison data where the network had older patients who were as likely to be Hispanic as the comparison population [15]. However, other studies have shown that Hispanic children are more likely to be uninsured and less likely to have a regular source of care and therefore may be less likely to be enrolled in a primary care network [25–28]. The finding of fewer 12–18 year olds in the primary care network is not surprising given the pattern of health care behavior among children. Younger children are more likely to present to a pediatrician's office for regular well child care, while adolescents, especially older adolescents, are less likely to present regularly for care. They are also more likely to be uninsured and to present to alternate sources of care [29–32]. A pediatrician's office may therefore not be the best place to capture a representative sample of adolescents within a community.

The other characteristic that was consistently different across most counties and neighborhoods was the receipt of Medicaid. The network children were generally more likely to receive Medicaid than nonnetwork children. This finding may be associated with insurance acceptance policies among the primary care practices within the study network compared to other practices in the region.

In multivariable analysis, we adjusted for neighborhood characteristics that could potentially affect the probability of enrollment in the primary care network. Our adjusted results showed little difference from our unadjusted results suggesting that the observed differences in enrolled and nonenrolled children were not confounded by such characteristics as the median income of neighborhood, average distance between subject's residence and practice sites, neighborhood household density, or presence of other pediatric providers.

Overall, our findings suggest that this primary care network could provide a representative sample to perform population-based surveillance particularly for the counties closest to CHOP and for the southern and western neighborhoods in Philadelphia. Based upon these findings, the primary care network may best represent younger children (<12 years of age) and non-Hispanic children. Of course, the important question is whether or not these characteristics are important epidemiologically for the potential infectious disease of interest and might influence the accuracy of infectious disease surveillance from the network. If a characteristic is important epidemiologically, this information could be used to adjust for differences between the CHOP and community population when measuring a particular disease. In this regard, it is interesting that pneumococcal disease rates were not significantly different between network and nonnetwork children, perhaps reflecting the fact that older children and Hispanic children are not particular risk groups for this disease, and therefore population estimates are not overly biased.

There are important limitations that may affect the interpretation of our results. A key issue is our use of the PHMC survey as a comparator database. While the survey is designed to provide a population-based sample, the weighted survey data did not agree with the US Census data results in terms of estimated population totals. For that reason, we poststratified the weights based upon census data so that the weighted sample would more closely resemble census results. Also, the PHMC survey sample size was relatively small compared to the primary care network sample, and, when stratified by neighborhood, the small number of PHMC subjects resulted in widely variable weights. Despite these limitations, the PHMC database does represent the only sample for the Philadelphia metropolitan region that contains both sociodemographic and clinical data.

We were unable to fully analyse 6.5% of our subjects due to inability to match their geocoded addresses to a neighborhood. However, this group was not significantly different from the remaining population across any of the measured characteristics. Therefore, we do not anticipate that their exclusion significantly affected our results. Another important consideration is generalizability. While this investigation is specific to one primary care network, the methodology may be applicable to other practice networks that use an integrated electronic medical record and whose patients reside in a region represented by a population-based sociodemographic database such as national census data. As more health systems within the US and other industrialized countries adopt integrated electronic medical records, this work could be generalizable to a wide range of communities across the US and internationally. In addition, our results demonstrated many general principles related to sampling that can be applied to any primary care or sentinel network seeking to obtain population estimates for disease based upon measures of the disease within their patient population. Lastly, we included only one indicator infectious disease, invasive pneumococcal disease, in our analysis. We selected this indicator condition because of the availability of reliable population-based surveillance data. Our results showed that the incidence of pneumococcal disease did not differ between network and nonnetwork patients, but our sample size was small and a proportion of our data was imputed.

Despite these limitations, we have established a framework for determining the ability for a pediatric primary care network to perform disease surveillance, particularly infectious diseases surveillance, within the communities served by the network. Our results show that this primary care network may be well served to provide disease estimates for the Philadelphia metropolitan region, particularly if known differences between the network and nonnetwork populations are accounted for. The estimates would be particularly robust for the counties and neighborhoods closest to the main hospital and practices within the network. This study has the potential to inform the development of a model that can be utilized to predict the prevalence and incidence of infectious diseases within the region served by a primary care network in order to perform surveillance of communicable diseases, antibiotic utilization patterns, antibiotic resistance, or potentially health service needs related to communicable disease control.

## Figures and Tables

**Table 1:** Demographic and clinical characteristics of the network-enrolled and the nonnetwork community populations in the philadelphia metropolitan area (*N* = 130,300 unweighted).

Characteristic	Network population *N* = 127,217% (S.E.)	Nonnetwork community population *N* = 3,083% (S.E.)	*P* ^a^
Gender			
Female	50.1 (0.14)	47.5 (1.08)	0.02
Race/ethnicity			
White	58.9 (0.15)	54.4 (1.08)	<.001
Black	37.7 (0.14)	24.0 (0.86)	
Asian	2.2 (0.04)	2.3 (0.53)	
Hispanic	2.2 (0.04)	19.4 (0.86)	
Age			
<1 year	8.7 (0.08)	6.8 (0.50)	<.001
1–5 years	36.2 (0.14)	26.3 (0.91)	
6–11 years	31.2 (0.13)	27.1 (0.90)	
12–18 years	24.0 (0.12)	39.7 (1.10)	
Diagnosis of asthma	16.6 (0.10)	16.3 (0.73)	n.s.
Diagnosis of diabetes	0.24 (0.01)	0.60 (0.84)	0.001
Receipt of Medicaid	28.0 (0.13)	19.3 (0.84)	<.001

^a^Rao Scott Chi square.

**Table 2:** Unadjusted and adjusted^a^ odds ratios for sociodemographic and clinical characteristics comparing network versus nonnetwork community population.

Characteristic	Unadjusted odds ratio (95% C.I.)	Adjusted odds ratio (95% C.I.)
Gender		
Female	1.10 (1.02, 1.21)	1.11 (1.02, 1.21)
Race/ethnicity		
White	1.00 (ref)	1.00 (ref)
Black	1.52 (1.38, 1.68)	1.91 (1.66, 2.20)
Asian	0.81 (0.51, 1.28)	0.75 (0.51, 1.09)
Hispanic	0.11 (0.10, 0.12)	0.14 (0.12, 0.16)
Age		
<1 year	1.00 (ref)	1.00 (ref)
1–5 years	1.09 (0.92, 1.29)	1.12 (0.93, 1.34)
6–11 years	0.91 (0.77, 1.08)	0.95 (0.80, 1.14)
12–18 years	0.48 (0.41, 0.57)	0.48 (0.40, 0.58)
Diagnosis of asthma	1.02 (0.92, 1.14)	1.02 (0.91, 1.14)
Diagnosis of diabetes	0.40 (0.24, 0.65)	0.48 (0.28, 0.82)
Receipt of Medicaid	1.61 (1.45, 1.79)	1.92 (1.70, 2.18)

^a^Weighted Logistic Regression adjusting for neighborhood average household size, average income of neighborhood, average distance traveled by patients within a neighborhood to their practice, and density of pediatric providers within residential neighborhood (Philadelphia County only).

## Abbreviations

GIS: Geographic information systems
CHOP: Children's Hospital of Philadelphia
PHMC: Philadelphia Health Management Corporation
MCD: Minor Civil Division.
